# Ivabradine Prevents Low Shear Stress Induced Endothelial Inflammation and Oxidative Stress via mTOR/eNOS Pathway

**DOI:** 10.1371/journal.pone.0149694

**Published:** 2016-02-18

**Authors:** Bing Li, Junxia Zhang, Zhimei Wang, Shaoliang Chen

**Affiliations:** Department of Cardiology, Nanjing First Hospital, Nanjing Medical University, 68^#^ Changle Road, Nanjing, 210006, China; The Chinese University of Hong Kong, HONG KONG

## Abstract

Ivabradine not only reduces heart rate but has other cardiac and vascular protective effects including anti-inflammation and anti-oxidation. Since endothelial nitric oxide synthase (eNOS) is a crucial enzyme in maintaining endothelial activity, we aimed to investigate the impact of ivabradine in low shear stress (LSS) induced inflammation and endothelial injury and the role of eNOS played in it. Endothelial cells (ECs) were subjected to LSS at 2dyne/cm^2^, with 1 hour of ivabradine (0.04μM) or LY294002 (10μM) pre-treatment. The mRNA expression of IL-6, VCAM-1 along with eNOS were measured by QPCR. Reactive oxygen species (ROS) was detected by dihydroethidium (DHE) and DCF, and protein phosphorylation was detected by western blot. It demonstrated that ivabradine decreased LSS induced inflammation and oxidative stress in endothelial cells. Western blot showed reduced rictor and Akt-Ser473 as well as increased eNOS-Thr495 phosphorylation. However, mTORC1 pathway was only increased when LSS applied within 30 minutes. These effects were reversed by ivabradine. It would appear that ivabradine diminish ROS generation by provoking mTORC2/Akt phosphorylation and repressing mTORC1 induced eNOS-Thr495 activation. These results together suggest that LSS induced endothelial inflammation and oxidative stress are suppressed by ivabradine via mTORC2/Akt activation and mTORC1/eNOS reduction.

## Introduction

The formation of atherosclerotic plaques is a complicated chronic process which involves not only inflammation of vessel walls but also endothelial dysfunction, as well as alteration of hemodynamic forces [1, 2]. Plaques are likely to form at bifurcations, vascular branches, inner curvatures and regions of stenosis where low and oscillatory blood flow cause damage to blood vessel wall [3, 4]. Laminar shear stress plays an very important role in protecting endothelial function, adjusting vascular tone, and protecting against the development of inflammation and atherosclerosis [5–7]. Studies have shown that the mechanical stimulation of shear stress can be transduced into intracellular signals by vascular endothelial cells (ECs) and finally resulting in eNOS activation [8, 9]. The mammalian target of rapamycin (mTOR) is an important protein in regulating protein synthesis, cell growth and proliferation, autophagy, cell metabolism, and stress responses [10]. Studies have indicated that the oscillatory flow activated PI3K/Akt/mTOR/p70S6K pathway may produce MG63 cell proliferation [11]. While the role mTOR pathway plays in LSS caused endothelial dysfunction and its relationship with eNOS is still unclear.

Ivabradine is a highly specific If-channel inhibitor that reduces heart rate by inhibiting pacemaker current in the sinus node [12]. In addition to heart rate reduction ivabradine has many other benefits such as anti-inflammation and anti-oxidation which were confirmed by clinical experiments and basic researches [13]. It has been proved that ivabradine prevents eNOS uncoupling and protects endothelial dependent vasodilatation [14].

Given all above, here comes to our hypothesis that LSS may induce endothelial injury and atherosclerosis by increasing inflammation and ROS generation, this process could be prevented by ivabradine via mTORC2/Akt and mTORC1/eNOS pathway.

## Materials and Methods

### Materials

The ivabradine, gelatin, protease inhibitor cocktail, LY294002, dimethyl sulfoxide (DMSO) and 4', 6-diamidino-2-phenylindole (DAPI) were obtained from Sigma (Saint Louis, Missouri, USA). Phosphatase inhibitor cocktail tablets were from Roche Applied Science (Mannheim, Germany). RIPA buffer and Reactive Oxygen Species Assay Kit were purchased from Beyotime (Nantong, China). BCA Protein Assay Kit and SDS-PAGE loading buffer were obtained from Keygen (Nanjing, China). Primary antibodies against GAPDH, phospho-eNOS, phospho-Akt, phospho-rictor, phospho-p70S6K, phospho-S6RP, phospho-raptor, IL-6, VCAM-1 and HRP-conjunct secondary antibody, Alexa Fluor® 488 conjugate anti-rabbit secondary antibody were purchased from Cell Signaling Technology (Beverly, MA, USA). Alexa Fluor® 488 conjugate goat anti-mouse secondary antibody was obtained from Thermo Fisher Scientific (Waltham, MA, USA). PVDF membrane and Immobilon western chemiluminescent HRP substrate were obtained from Millipore (Billerica, MA, USA). ROS fluorescent probe-DHE was from Vigorous Biotechnology (Beijing, China). TRIzol was purchased from Invitrogen (Grand Island, NY, USA). PrimeScript RT reagent Kit and SYBR Premix Ex Taq were from TakaRa Biotechnology (Dalian, China).

### Cell culture

Human umbilical vein endothelial cells (HUVECs) were obtained from the Type Culture Collection of the Chinese Academy of Sciences (Shanghai, China) and grown in RPMI medium 1640 (Gibco) supplemented with 10% fetal bovine serum (Hyclone). After incubated at 37°C with 5% CO_2_ and reaching 90% confluence, the cells were untouched and seeded onto glass slides (30×50 mm) that had been pre-coated with 1% gelatin for at least 16 hours before the flow study was undertaken. In some experiments HUVECs were pre-treated with ivabradine (0.04μM) or LY294002 (10μM) which was diluted in a FBS-free culture medium for 1 hour before and during the flow study. The control group cells which were pre-seeded on the glass slides had been cultured in serum free medium for 1 hour to match the experimental groups.

### Laminar fluid flow appliance

A parallel plate flow chamber was used to supply laminar fluid flow. In brief, the chamber was made-up of two stainless steel plates with a silicon gasket between them, a cell-seeded glass slide could be placed exactly into the cuboid groove in the middle of the plate. A liquid accumulator that containing a temperature-control device was linked with the chamber, 5% CO_2_ was continuously gassed into it to keep fluid pH steady at 7.4. Fluid shear stress generated on endothelial cells can be estimated by a pressure transducer connected to the chamber. Flow pre- and after-load were regulated to control the shear stress value. All parts of the flow appliance were sterilized prior to the flow study. The following parameters were used in this study: flow chamber height = 56mm, pump rate = 60 times/minute, flow shear stress was 2 dynes/cm^2^. After the above preparations were completed the glass slides with endothelial cells were put into parallel plate flow chamber and 250ml serum-free 1640 medium was added into the liquid accumulator as cycle fluid. In some experiments FBS-free 1640 with ivabradine (0.04μM) or LY294002 (10μM) were used as cycle fluid. Different time periods of laminar flow (0, 5, 15, 30, 60, 120 minutes) were applied to cells. Then total RNA was collected to detect IL-6, VCAM-1 and eNOS changes on gene expression caused by LSS, while proteins in cytoplasm were obtained for cell signal pathway investigation.

### Real-time quantification polymerase chain reaction (QPCR)

After being exposed to low shear stress of 2 dynes/cm^2^, the endothelial cells on glass slides were washed twice with ice cold phosphate buffered saline (PBS). The total RNA was isolated using TRIzol. cDNA was generated by using the PrimeScript RT reagent Kit, then it was amplified according to the instructions of the SYBR Premix Ex Taq kit by using an ABI7500 system (Grand Island, NY, USA). Three parallel samples were performed for each cDNA, and human glyceraldehyde-3-phosphate dehydrogenase (GAPDH) was used as an internal control. IL-6, VCAM-1 and eNOS expressions were measured after 30 and 120 minutes’ LSS exposure and compared with control cells. In some groups cells were pre-treated with ivabradine or LY294002 for 1 hour before being exposed to LSS. Thereafter IL-6, VCAM-1 and eNOS mRNA expressions were examined. Primer sequences for human GAPDH are 5’-GACCTGACCTGCCGTCTA-3’ (forward) and 5’-AGGAGTGGGTGTCGCTGT-3’ (reverse), for human endothelial nitric oxide synthase (eNOS) they are 5’-TGTTTCTGTCTGCATGG-3’ (forward) and 5’-TGGCTGGTAGCGGAAGG-3’ (reverse), for human IL-6 they are 5’-TCGAGCCCACCGGGAACGAA-3’ (forward) and 5’-GCAACTGGACCGAAGGCGCT-3’ (reverse), for human VCAM-1 they are 5’-GACTTGCAGCACCACAGGCT-3’ (forward) and 5’-TCTCCAGCCGGTCAAGGGGG-3’ (reverse).

### Immunofluorescence (IF)

The expression of IL-6, VCAM-1 and eNOS-Thr495 were detected by immunofluorescence (IF). After LSS applied endothelial cells were washed twice with ice cold PBS then fixed in 4% paraformaldehyde for 20 minutes. Glass slides with fixed cells were washed once in PBS for 5 minutes at room temperature on a shaker. Then cells were washed with PBST (PBS with 0.2% Triton-100) for 5 minutes to raise the permeability. After that cells were washed again with PBS followed with incubation of goat serum for 1 hour to block non-specific antigens in a humidified chamber. The primary antibodies were diluted in goat serum and incubated with cells overnight at 4°C in a humidified chamber. Then cells were washed twice with PBST along with PBS washed for once. The secondary antibodies were also diluted in goat serum and incubated at room temperature in a dark humidified chamber for 2 hours. Thereafter cells were again washed twice with PBST along with PBS washed for once. Nuclei were detected by DAPI. Fluorescence microscope (Olympus, Japan) was used to observe positive cells with bright green fluorescence.

### ROS detection by DHE

Dihydroethidium (DHE) was used to detecte ROS generation in HUVECs. DHE is a cell-permeant reagent that intercalates within the cell’s DNA, staining its nucleus a bright fluorescent red when it is oxidized by ROS. Briefly, after being exposed to shear stress for 0, 30 and 120 minutes endothelial cells were washed gently with ice cold PBS twice and then incubated in 5 μM DHE at 37°C for 20 minutes in a dark environment. Thereafter the cells were washed with PBS twice to remove DHE that did not combine with nucleus, and were incubated with DAPI for 10 minutes to detect nucleus. After that, cells were washed with PBS twice and observed under a fluorescent microscope (Olympus, Japan). Cell that had bright fluorescent red in its nuclei was defined as ROS-positive. DAPI-positive cells that stained into bright blue were counted as total cell number. The positive rate was expressed as ROS-positive cell number versus total cell number. At least 5 different visual field images per group were used for statistics. In some groups, cells were pre-treated with ivabradine or LY294002 for 1 hour before shear stress applied, then ROS was detected.

### ROS detection by DCFH-DA

DCFH-DA is a non-fluorescent and cell- permeant reagent. After enter into cells DCFH-DA can be hydrolyzed to DCFH by esterase. Intracellular ROS generation oxidizes non-fluorescent DCFH to fluorescent DCF which can be detected by fluorescence microscope. Hence, the rate of DCFH oxidized to DCF can be represented as the quantity of ROS. Endothelial cells that exposed to LSS with or without ivabradine pre-treatment were washed twice with ice cold PBS. Then 10μM/L DCFH-DA was added onto cells and incubated at 37°C for 20 minutes in a dark environment. After that, cells were washed twice with PBS to remove probes that did not combine. Cells were then incubated with DAPI to detect the nucleus. Thereafter, extra DAPI was washed out with PBS and cells were observed under a fluorescent microscope (Olympus, Japan). DCF-positive were defined as cells that had bright fluorescent in their cytoplasm. DAPI-positive cells were defined the same as previous. The positive rate was expressed as DCF-positive cell number versus total cell number.

### Western blot analysis

Proteins in cells that exposed to LSS for different time period (0, 5, 15, 30, 60, 120 minutes) and cells that pre-treated with ivabradine and LY294002 before LSS applied were collected. Glass slides with cells were washed gently twice with ice cold PBS and lysed with a buffer containing RIPA, 10% phosphatase inhibitor and 1% proteinase inhibitor. Lysis buffer was centrifuged at 12000rpm, at 4°C for 20 minutes, the supernatants with protein were collected. The BCA Protein Assay Kit was used to determine protein concentrations. In general, equal amounts of protein were separated on 8% SDS-PAGE and transferred to PVDF membranes, then the membranes were incubated in non-fat milk for 1 hour at room temperature. After that, PVDF membranes were washed 3 times in TBST for 10 minutes each and incubated in non-fat milk diluted primary antibodies at 4°C overnight. Next, the membranes were washed in TBST followed by incubating with HRP-conjugated secondary antibody for 2 hours at room temperature. Immobilon western chemiluminescent HRP substrate was applied to detect the signals and Image J was used to analysis intensity of each group.

### Statistical analysis

Data were expressed as means ± standard deviation (SD). One way ANOVA was used to compare data that come from more than two groups. Analyses were performed using SPSS version 21.0 (IBM Corporation, Armonk, USA), and P values less than 0.05 were considered statistically significant.

## Results

### Ivabradine attenuated LSS-induced IL-6 and VCAM-1 mRNA expression in HUVECs

**Fig 1:**
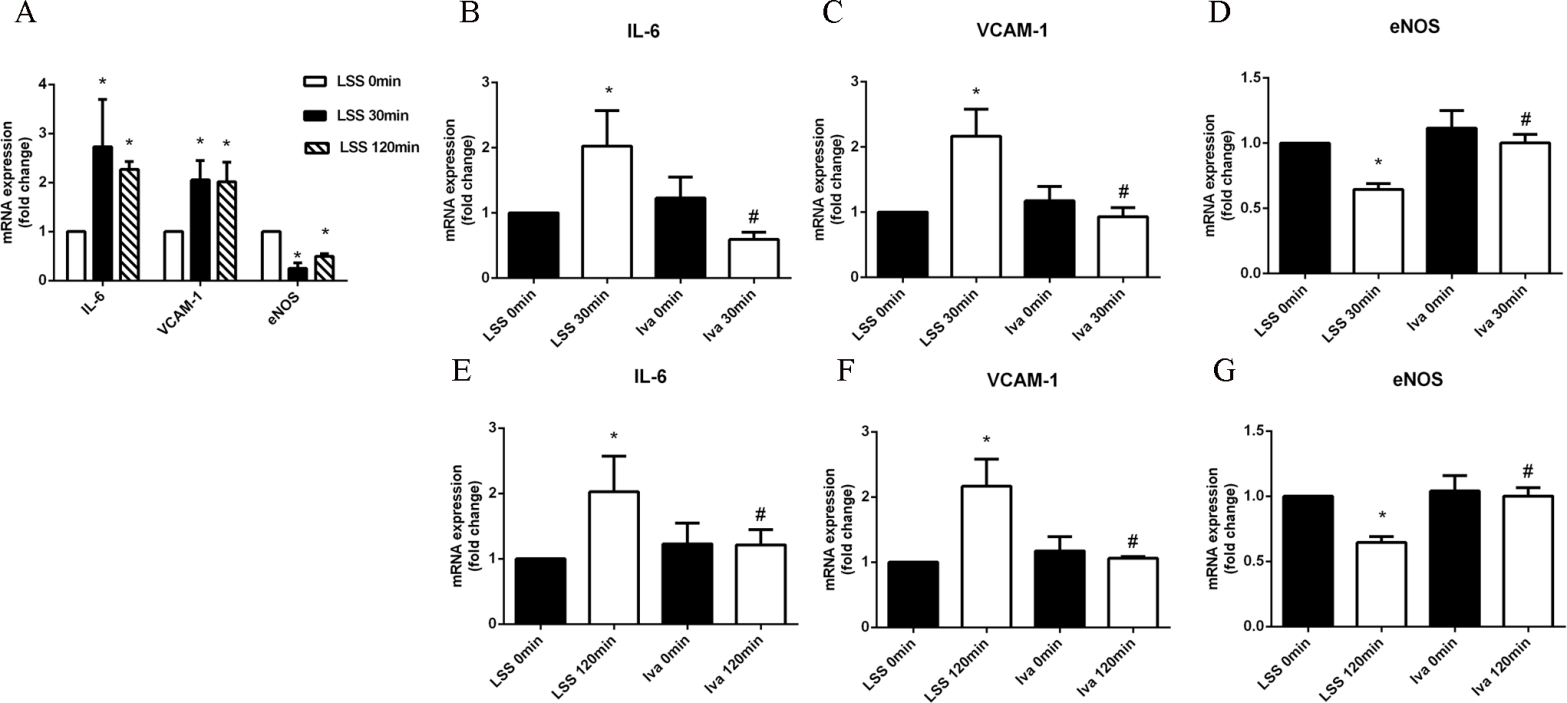
LSS induced changes of anti-/pro- inflammatory factors. (A) mRNA expression of IL-6, VCAM-1 and eNOS after LSS was applied for 0, 30 and 120 minutes. (B-G) Ivabradine reverses LSS caused mRNA alteration of IL-6, VCAM-1 and eNOS. All experiments above were repeated three times.

**Fig 2:**
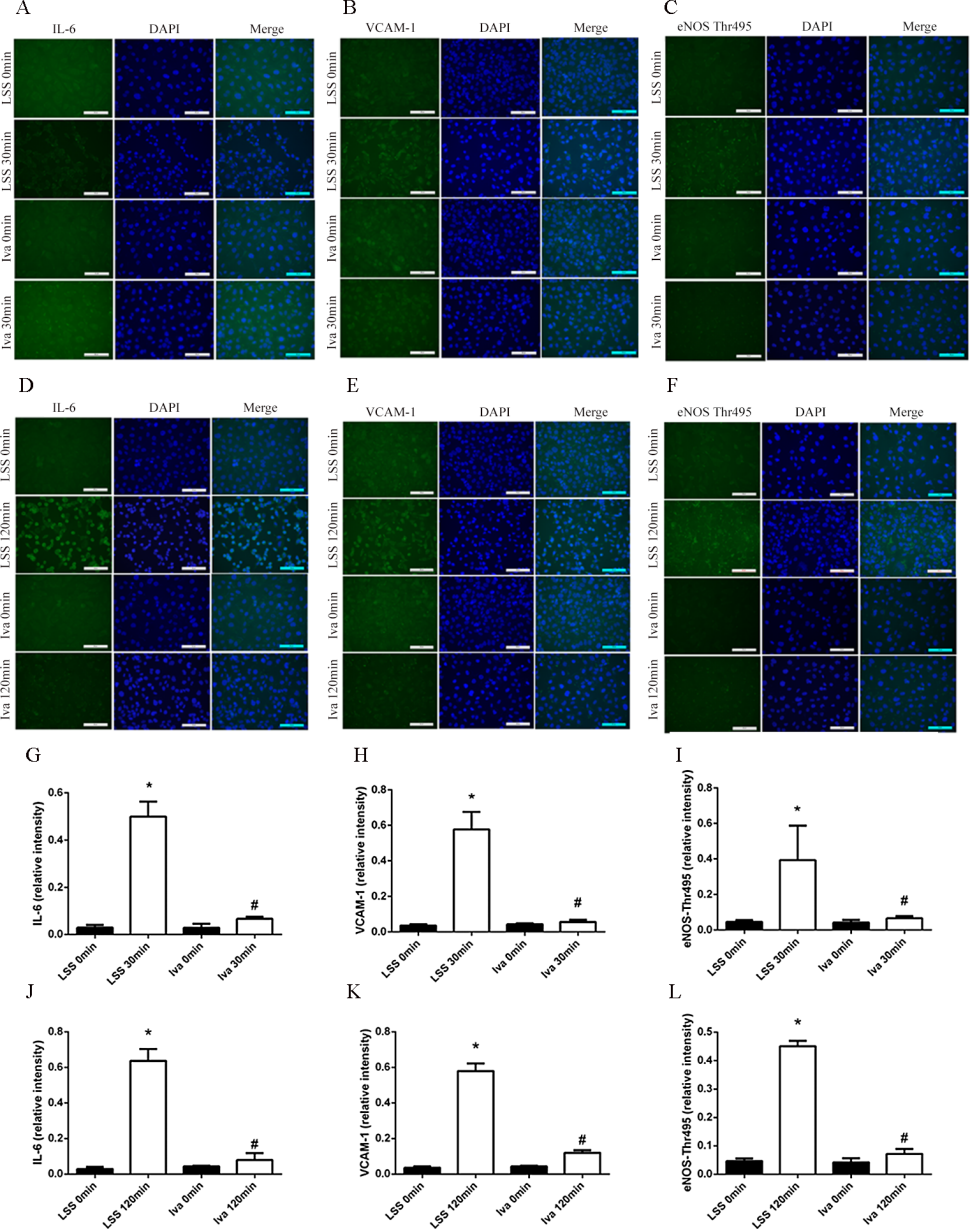
Immunofluorescence of IL-6, VCAM-1 and eNOS-Thr495 generated by LSS. (A-F) Representative images of immunofluorescence. Positive cells in bright green were counted for five different visual fields. (G-L) Bar diagram showing quantitative data of positive cells. *p<0.05 versus LSS 0 minute, # p<0.05 versus LSS 30 or 120 minutes.

HUVECs were exposed to LSS, then the total RNA was collected and cDNA was generated. The expressions of IL-6, VCAM-1 and eNOS were measured by real-time PCR and ct values of each sample were compared statistically using the 0 minute group as control. As shown in Fig 1A, LSS applied for 30 and 120 minutes dramatically increased the mRNA expression of IL-6 and VCAM-1, while it decreased eNOS expression. As shown in Fig 1B to 1G, ivabradine pre-treatment decreased LSS induced IL-6 and VCAM-1 expression, while it increased eNOS expression. In addition, ivabradine alone did not change the amount of mRNA expression. Immunofluorescence of IL-6, VCAM-1 and eNOS-Thr495 confirmed the results above. The expression of IL-6, VCAM-1 and eNOS-Thr495 in cytoplasm were raised by LSS, and ivabradine attenuated them almost to basal line (Fig 2). Since eNOS was the most important enzyme for the release of the vascular relaxation agent NO, these results indicate that ivabradine attenuated LSS induced inflammation by increasing eNOS expression.

### Ivabradine decreased LSS induced ROS generation in HUVECs

**Fig 3:**
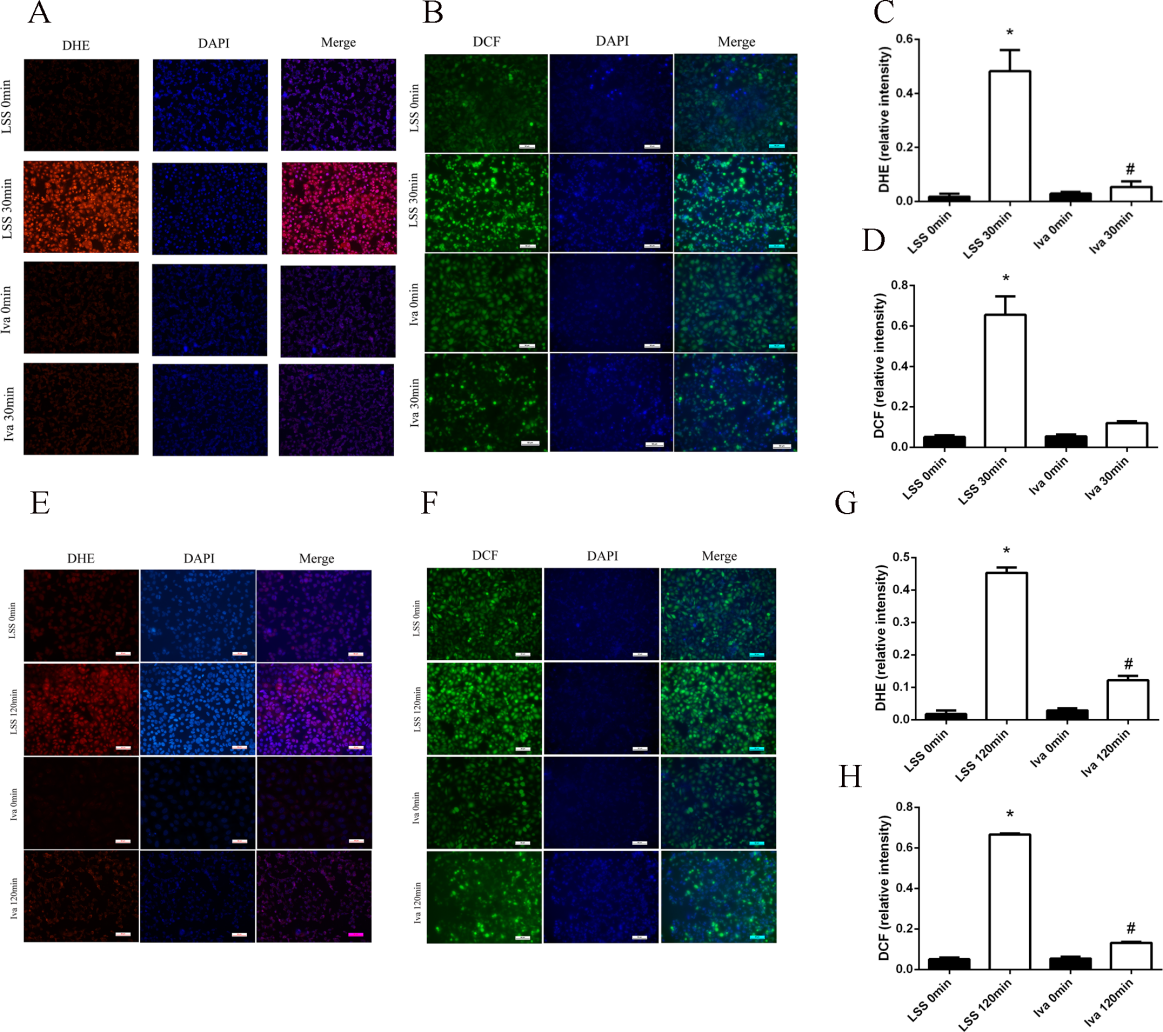
Fluorescence microscopy of ROS-positive cells stained with DHE and DCF and nucleus stained with DAPI. (A, E) DHE-positive cells in red were counted for five different visual fields. (B, F) DCF-positive cells in bright green were counted for five different visual fields. (C, G) Bar diagram showing quantitative data of DHE-positive cells. (D, H) Bar diagram showing quantitative data of DCF-positive cells. *p<0.05 versus LSS 0 minute, # p<0.05 versus LSS 30 or 120 minutes.

DHE and DCFH-DA were used to detect ROS generation in endothelial cells that had been exposed to LSS with or without pre-treatment of ivabradine for 1 hour. Compared with cells in static (LSS 0 minute), 30 minutes of LSS exposure remarkably elevated the ROS concentration. However, ivabradine brought it down almost to the control level when it alone barely had any influence on ROS generation (Fig 3A to 3D). LSS applied for 120 minutes also significantly increased ROS generation in ECs and after ivabradine pre-treatment ROS was reduced. (Fig 3E to 3H). This result suggested that ivabradine preserved endothelial injury by reducing LSS caused oxidative stress.

### LSS induced endothelial inflammation and oxidative stress via activation of mTORC1/eNOS-Thr495 and inhibition of mTORC2/Akt

**Fig 4:**
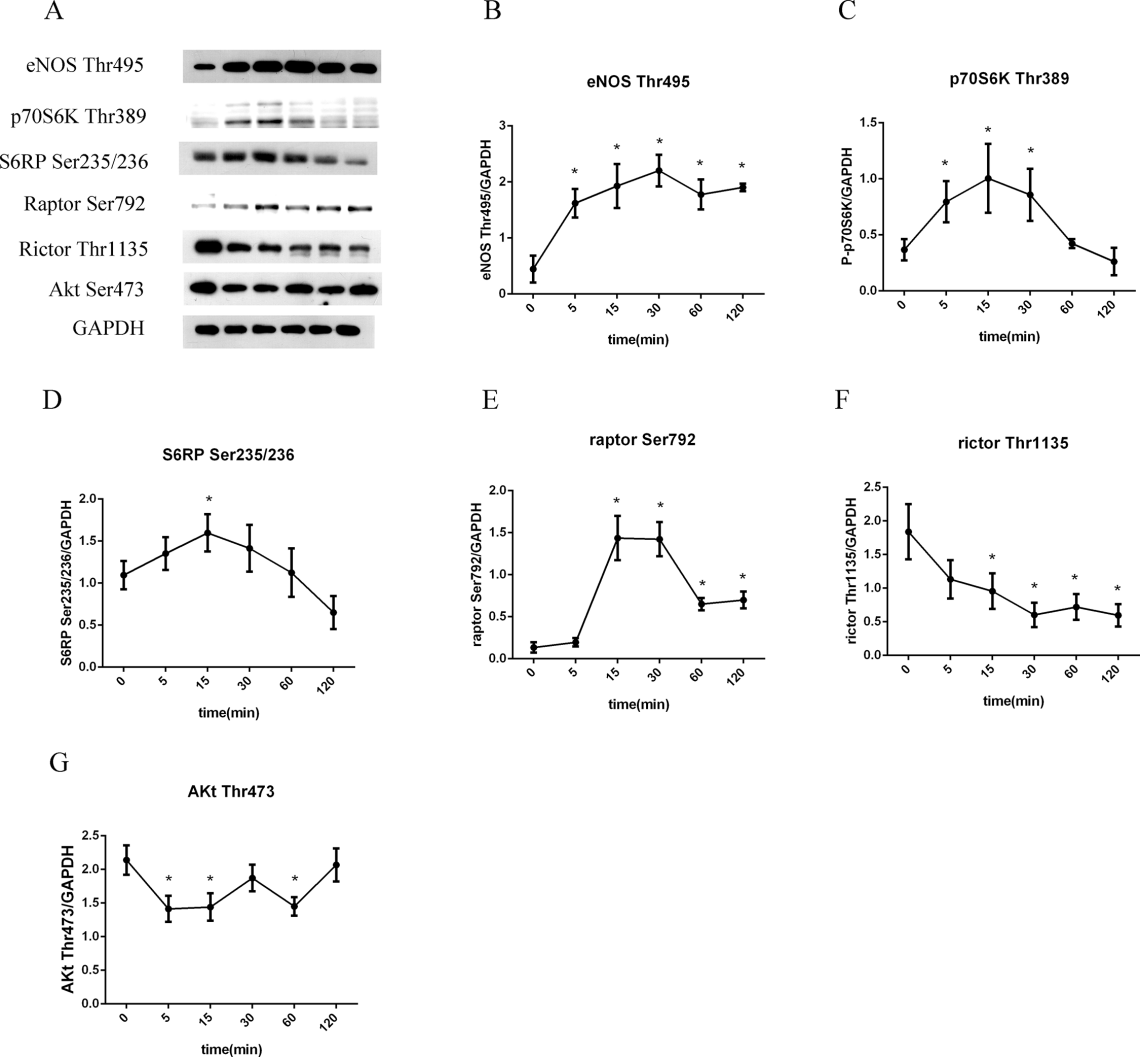
Effect of LSS on eNOS and mTOR pathway. (A) Representative western blot images of eNOS-Thr495, p70S6K-Thr389, S6RP-Ser235/236, raptor-Ser792, rictor-Thr1135, Akt-Ser473 and GAPDH that exposed to LSS for time periods. (B-G) Line charts showing intensity of western blot images, all data were normalized by GAPDH. *p<0.05 versus LSS 0 minute.

To find out the signal pathways that may be involved in LSS induced endothelial inflammation and oxidative stress, we then exposed HUVECs to LSS for different time periods. Results showed that LSS ascended eNOS-Thr495 activity in a time dependent manner, which started after only 5 minutes’ exposure and with a prolonged effect for at least 120 minutes (Fig 4A and 4B). We next determined the expression of mTOR pathway phosphorylation state. As shown in Fig 4C to 4E, LSS induced p70S6K-Thr389 and S6RP-Ser235/236 expression in 5 minutes and this continued for 30 minutes, which was in accordance with the changes of raptor-Ser792. However, the expression of these three proteins began to drop when LSS applied for more than 30 minutes. As for mTORC2 pathway, LSS decreased rictor-Thr1135 and Akt-Ser473 phosphorylation as time passed, it initiated after 5 minutes, and lasted for 120 minutes (Fig 4F and 4G).

Taken together, we believed that within 30 minutes LSS led to endothelial inflammation and oxidative stress by activation of mTORC1/eNOS-Thr495 and inhibition of mTORC2/Akt pathway. When exposure time prolonged, eNOS-Thr495 was mainly activated by inhibition of mTORC2/Akt pathway and mTORC1 was no longer involved. As mTORC1 expression was changed after LSS was applied for 30 minutes, 30 and 120 minutes were chosen for subsequent experiments.

### Ivabradine reversed LSS caused endothelial injury by altering expression of mTOR pathway and eNOS-Thr495 phosphorylation

**Fig 5:**
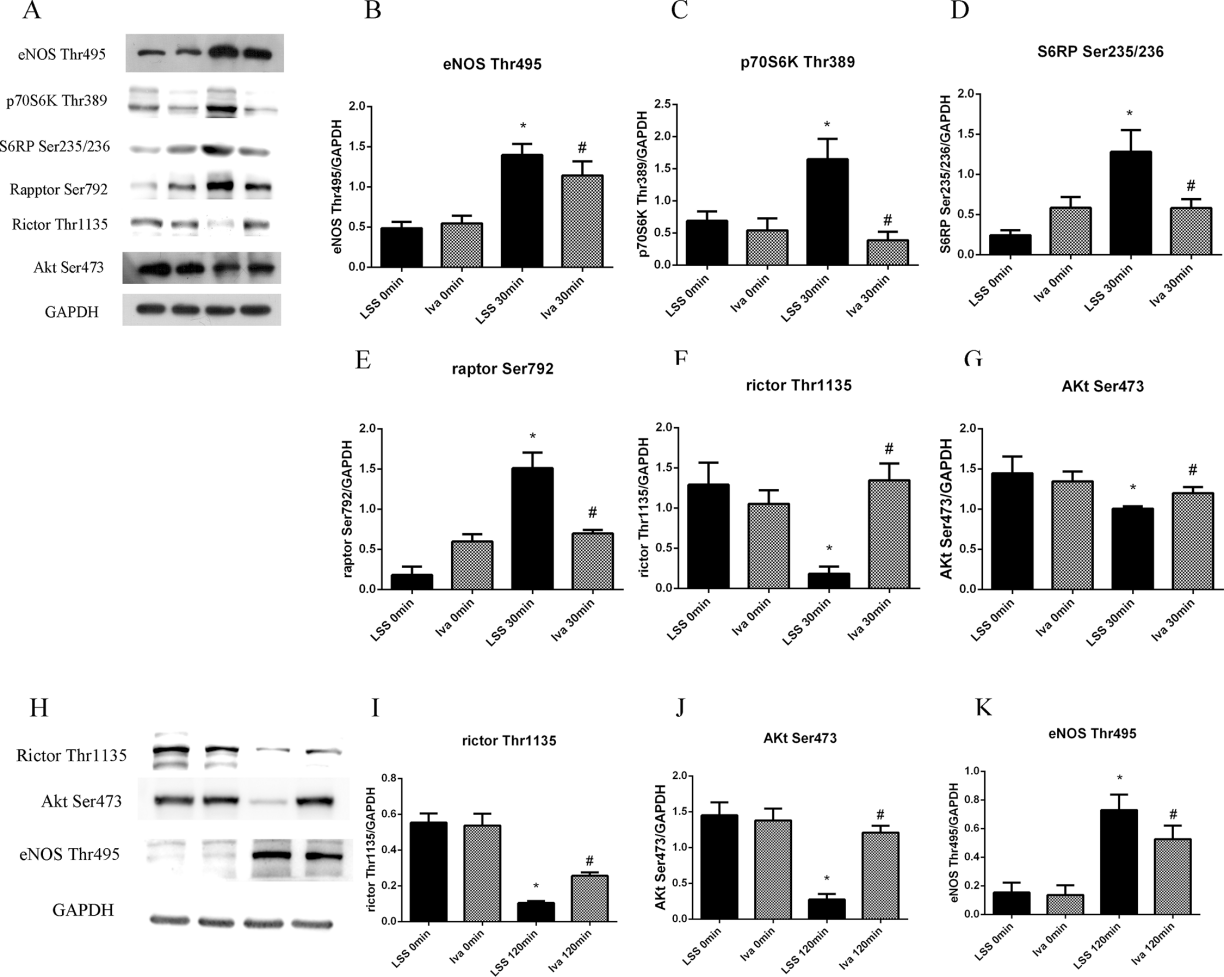
Effect of ivabradine on LSS induced changes of eNOS and mTOR pathway. (A) ECs were exposed to LSS for 30 minutes after pre-treated with ivabradine, protein phosphorylation was compared in cells exposed to LSS with or without pre-treatment of ivabradine. (B-G) Bar diagram showing intensity data of western blot images, all data were normalized by GAPDH. (H) mTORC2 pathway expression in cells exposed to LSS for 120 minutes after pre-treated with ivabradine. (I-K) Bar diagram showing intensity data of western blot images, all data were normalized by GAPDH. *p<0.05 versus LSS 0 minute, # p<0.05 versus LSS 30 or 120 minutes.

To investigate the mechanism of ivabradine improved endothelial function, cells were pre-incubated with ivabradine for 1 hour before being exposed to LSS. Fig 5A and 5B showed that after 30 minutes exposure ivabradine reversed LSS produced eNOS-Thr495 phosphorylation prominently, yet without influence on control cells. Possible effects of ivabradine on mTOR pathway were examined next. Fig 5C to 5E showed that phosphorylation of raptor, p70S6K and S6RP which aroused by LSS were reduced by ivabradine. It was also shown that ivabradine neutralized the effects of LSS and increased the activity of mTORC2/Akt pathway (Fig 5F and 5G). Ivabradine also raised mTORC2/Akt phosphorylation in cells exposed to LSS for 120 minutes (Fig 5H to 5K). All of above suggests that when LSS applied within 30 minutes ivabradine exerts endothelial protective effect through elevating mTORC2/Akt and attenuating mTORC1/eNOS-Thr495 expression, when LSS exposure lasts ivabradine protects endothelial mainly by increasing mTORC2/Akt pathway. As mTORC2/Akt expression had achieved stability when LSS was applied for 30 minutes, this time point was chosen for subsequent experiments.

### Ivabradine reduced mTORC1/eNOS-Thr495 by rising mTORC2/Akt

**Fig 6:**
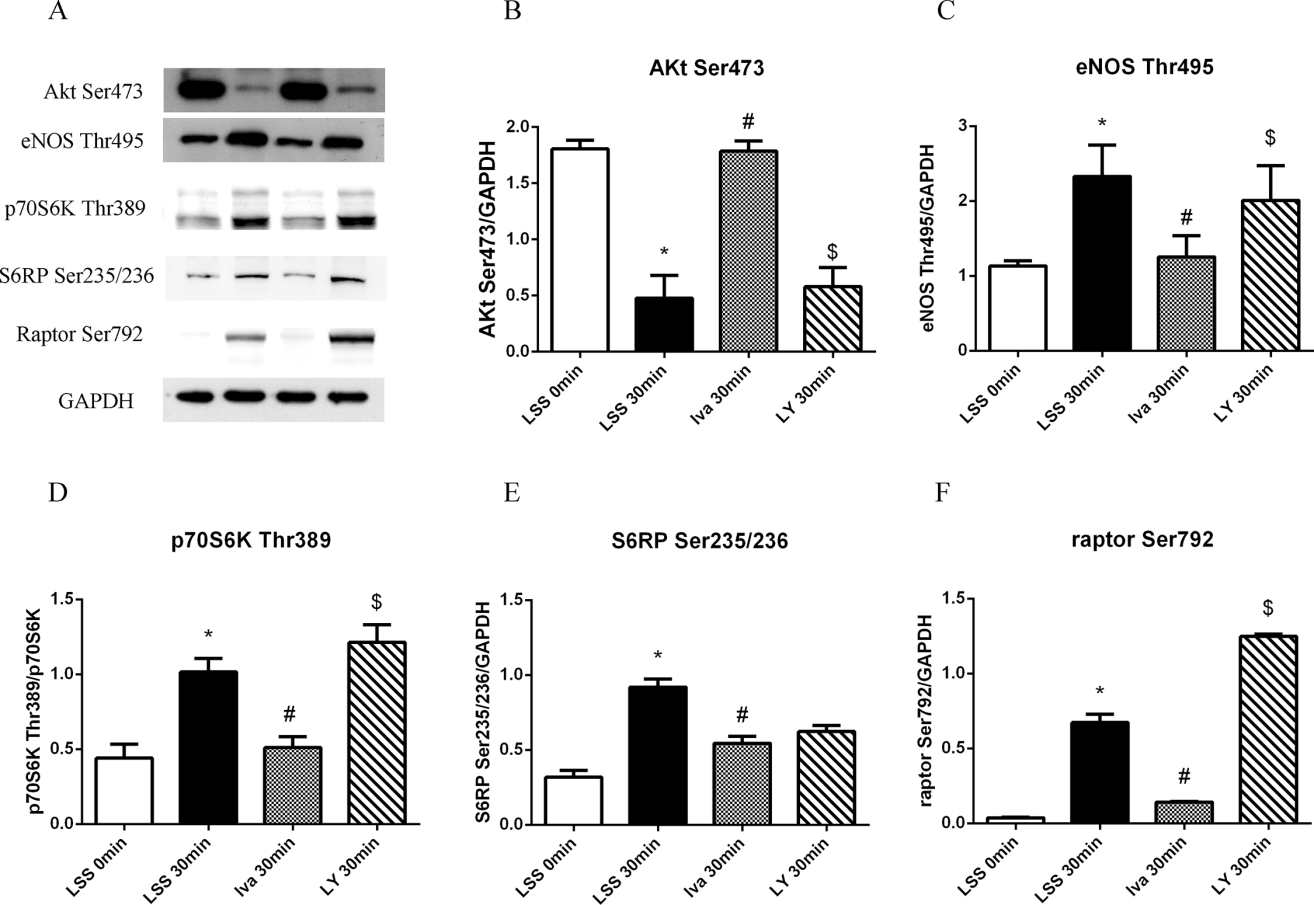
Effect of LY294002 on eNOS and mTOR pathway. (A) LY294002 was applied before ivabradine pre-treatment and LSS exposure, protein phosphorylation was compared in cells exposed to LSS for 30 minutes with or without ivabradine pre-treatment. (B-F) Bar diagram showing intensity data of western blot images, all data were normalized by GAPDH. *p<0.05 versus LSS 0 minute, # p<0.05 versus LSS 30 minutes, $p<0.05 versus Iva 30 minutes.

To explore whether there is a connection between mTORC1/eNOS-Thr495 and mTORC2/Akt in ivabradine induced endothelial protection, a specific inhibitor of Akt was used. We pre-treated HUVECs with LY294002 before ivabradine treatment. LY294002 effectively suppressed Akt-Ser473 expression almost down to the levels seen after 30 minutes’ of LSS exposure compared with ivabradine treated alone (Fig 6A and 6B). Then we tested the impact of LY294002 on eNOS-Thr495 generation and it turned out that eNOS phosphorylation at Thr495 had declined (Fig 6C). This meant abolished endothelial protection and ROS suppression by ivabradine. The effect of Akt inhibitor on mTOR pathway was also detected. LY294002 impaired ivabadine induced mTORC1 reduction revealing as increased raptor, p70S6K and S6RP phosphorylation (Fig 6D to 6F).

To sum up, ivabradine prevented endothelial cells from being impaired by LSS via blocking the signal from mTORC1 to downstream protein eNOS-Thr495, which was achieved by arousing mTORC2/Akt pathway, so that NO release was preserved and ROS generation was reduced.

### LY294002 neutralized the endothelial protective effect of ivabradine

**Fig 7:**
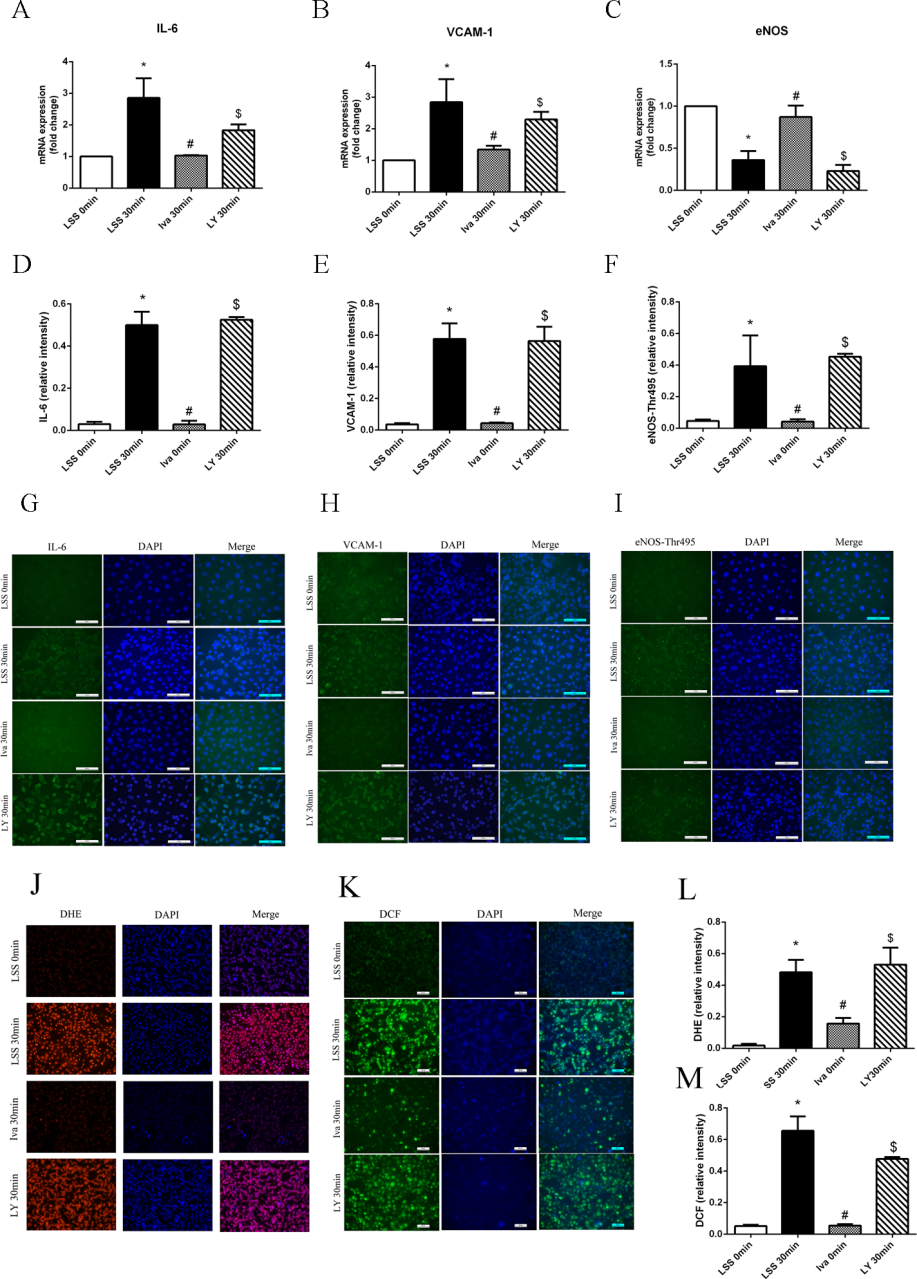
Effect of LY294002 on expression of anti-/pro- inflammatory factors and ROS generation. (A-C) mRNA expression of IL-6, VCAM-1 and eNOS after LSS was applied with or without LY294002 pre-treatment compared with ivabradine pre-treated cells. (D-F) Bar diagram showing quantitative data of immunofluorescence positive cells. Cells in bright green were counted for five different visual fields. (G-I) Immunofluorescence of IL-6, VCAM-1 and eNOS-Thr495 generated by LSS. (J-K) LY294002 was applied before ivabradine pre-treatment and LSS exposure, ROS generation was compared within groups. (L-M) Bar diagram showing quantitative data of ROS-positive cells. *p<0.05 versus LSS 0 minute, # p<0.05 versus LSS 30 minutes, $p<0.05 versus Iva 30 minutes.

As shown in Fig 7A and 7B, LY294002 pre-treatment remarkably elevated IL-6 and VCAM-1 mRNA generation compared with cells treated with ivabradine alone. Also Akt inhibition decreased eNOS mRNA expression almost down to as much as in cells exposed to LSS for 30 minutes (Fig 7C). Immunofluorescence of IL-6, VCAM-1 and eNOS-Thr495 showed the same results as above (Fig 7D to 7I). ROS was detected in LY294002 pre-treated cells, which shows that ROS generation was raised even if the cells were also pre-treated with ivabradine (Fig 7J to 7M). All these findings together suggest that Akt played an important role in endothelial protective effect of ivabradine.

## Discussion

The present study suggests that there is a novel signal pathway that links ivabradine and LSS induced endothelial inflammation and oxidative stress. It shows that ivabradine suppresses LSS caused eNOS-Thr495 phosphorylation via up-regulation of mTORC2/Akt expression which in turn down-regulates mTORC1/eNOS-Thr495 expression.

Shear stress is a crucial mechanical force applied on endothelial cells that affects the structure and biological function of vascular endothelium [15, 16]. Many researches have been done on laminar shear stress. However, it is low and disturbed shear stress that play the main part in the development of atherosclerosis [6, 11, 17]. In the current study we found that endothelial function was affected when LSS was applied. It was manifested as an increased expression of inflammatory molecules (IL-6 and VCAM-1) and decreased anti-atherosclerosis factor eNOS. This is consistent with the previous reports of Stéphanie Lehoux [6, 18]. Then we investigated the signal molecules that are responsible for endothelial injury caused by LSS. It turned out that eNOS-Thr495 expression was raised after shear stress application which suppressed the release of endothelial generated NO. Since shear stress is not a chemical molecule that can directly transduce signals to eNOS by receptors, we believe that there must be a pathway that helps transfer the signal instead [15]. Many studies have proved that the mTOR pathway is closely related to cell inflammation and apoptosis since it transmits signals from the cytoplasm into the cell nucleus altering the activity of transcription factors which finally leads to changes of gene expression [19, 20]. It is known that in order to exert its function mTOR has to combine with specific adaptor proteins to form the mTOR complex 1 (mTORC1) and mTOR complex 2 (mTORC2), so the amounts of raptor and rictor were detected. We observed elevated raptor phosphorylation as well as p70S6K and S6RP which were downstream of mTORC1 pathway. Also we discovered attenuated rictor and Akt-Ser473 phosphorylation which was known as downstream of mTORC2 pathway. All of above indicate that LSS injures endothelial via arousing mTORC1/eNOS-Thr495 and decreasing mTORC2/Akt.

Ivabradine is a bradycardic without negative inotropic effects which favors patients with heart failure and angina [21–23]. Previous clinical trials have demonstrated that patients benefit from ivabradine in relation to its heart rate reduction effect [24, 25]. BEAUTIFUL (morbidity-mortality evaluation of the If inhibitor ivabradine in patients with coronary disease and left ventricular dysfunction) study showed ivabradine reduces mortality and cardiovascular events in patients with stable coronary artery disease and left ventricular systolic dysfunction only when their heart rate were under 70bpm [25]. Yet we have found that ivabradine has anti-inflammatory and anti-oxidant effects independent of heart rate reduction in protecting endothelial from being impaired by LSS. This result is supported by Thomas Walcher et al, who indicated that ivabradine reduced CD4-positive lymphocyte migration via inhibition of chemokine-induced PI3K/Akt activity [26]. In order to determine the effect of ivabradine on LSS induced endothelial impairment, cells were pre-treated with ivabradine before LSS was applied. We observed decreased raptor, p70S6K and S6RP phosphorylation which revealing the inhibition of mTORC1 pathway when ECs were exposed to LSS within 30 minutes. Meanwhile, the eNOS-Thr495 expression was decreased as time went on for at least 120 minutes. It also showed increased rictor phosphorylation in ivabradine pre-treatment group compared with LSS applied alone. As the downstream of mTORC2 pathway, Akt-Ser473 expression was elevated too. It seemed ivabradine protected endothelial in two different ways. However, studies have shown that mTORC1 and mTORC2 are connected sometimes. The relationship between mTORC1 and mTORC2 in the endothelial protective effect of ivabradine is unclear, but it seems certain that they are both very important in maintaining cardiac homeostasis [10]. By using the Akt inhibitor LY294002, we found that ivabradine inhibited mTORC1/eNOS-Thr495 activity was caused by the enhancement of mTORC2/Akt pathway. This view is supported by the findings of Völker et al that mTORC2 suppressed mTORC1 activity by stimulating Akt phosphorylation and cardiac function was thereby preserved after myocardial infarction [27]. Searches on mTORC1 are much clearer than that of mTORC2. It seems that mTORC1 is more sensitive to stress than mTORC2. Yet, when responding to stress, mTORC1 is usually activated along with its upstream Akt-Thr308 [10]. The situation in our experiment is quite different since Akt-Ser473 is known as the downstream of mTORC2. Besides, after LY294002 pre-treatment the anti-inflammatory effect of ivabradine was neutralized as well as the elevated mTORC2/Akt pathway, which confirmed that mTORC2/Akt pathway was upstream of mTORC1 and eNOS in ivabradine preserved endothelial from injured by LSS.

There are some limitations in this study. Firstly, we do not find out the exact receptor which directly transduces the force of LSS into cytoplasm. By discovering this exact receptor, a specific inhibitor might be produced to prevent endothelium from being injured and the process of atherosclerosis formation could be delayed. Secondly, the real shear stress applied on atherosclerosis vessels is much more complicated and uncontrollable than what we use in this study. In vivo experiments should be conducted to confirm our findings. Briefly, much more work is needed to clarify the relationship between ivabradine and LSS induced endothelial dysfunction.

## Conclusion

Our results demonstrate that ivabradine preserves endothelial from LSS induced inflammation and oxidative stress and postpones the progress of plaque formation via altering eNOS activity. And this effect is mediated by mTORC2/Akt pathway which passes on the signal to mTORC1 and acts on eNOS at last. Hopefully, our study would help find new targets for treatment of coronary artery disease.

## Supporting Information

S1 Table Data for QPCR. (PDF)

S2 Table Data for immunofluorescence and ROS detection. (PDF)

S3 Table Data for western blot. (PDF)
