# Supplementary material for: Ivabradine Prevents Low Shear Stress Induced Endothelial Inflammation and Oxidative Stress via mTOR/eNOS Pathway
Source: PLoS One. 2016 Feb 18;11(2):e0149694. doi: 10.1371/journal.pone.0149694 (PMC4758626; doi:10.1371/journal.pone.0149694)
Supplement: S1 Table — (PDF) [file pone.0149694.s001.pdf]

| $2^{-\Delta\Delta CT}$ / group | IL-6 | VCAM-1 | eNOS |
|---|---|---|---|
| LSS0 | 1 | 1 | 1 |
| LSS0 | 1 | 1 | 1 |
| LSS0 | 1 | 1 | 1 |
| LSS30 | 3.802636 | 2.396618 | 0.118503 |
| LSS30 | 2.45207 | 2.142062 | 0.289975 |
| LSS30 | 1.943962 | 1.626758 | 0.334946 |
| LSS120 | 2.139094 | 2.105262 | 0.552099 |
| LSS120 | 2.212995 | 1.573525 | 0.489032 |
| LSS120 | 2.450371 | 2.361985 | 0.443806 |

| $2^{-\Delta\Delta CT}$ / group | IL-6 | VCAM-1 | eNOS |
|---|---|---|---|
| LSS0 | 1 | 1 | 1 |
| LSS0 | 1 | 1 | 1 |
| LSS0 | 1 | 1 | 1 |
| LSS30 | 1.143138 | 3.069492 | 0.819605 |
| LSS30 | 1.598812 | 2.472551 | 0.737646 |
| LSS30 | 1.302244 | 2.194664 | 0.857971 |
| Iva0 | 1.06437 | 1.095812 | 1.059953 |
| Iva0 | 0.964598 | 1.006956 | 0.950659 |
| Iva0 | 1.044636 | 0.993093 | 0.968618 |
| Iva30 | 0.629379 | 0.533663 | 0.992404 |
| Iva30 | 0.46684 | 0.382094 | 0.988286 |
| Iva30 | 0.68302 | 0.262611 | 1.032399 |

| $2^{-\Delta\Delta CT}$ / group | IL-6 | VCAM-1 | eNOS |
|---|---|---|---|
| LSS0 | 1 | 1 | 1 |
| LSS0 | 1 | 1 | 1 |
| LSS0 | 1 | 1 | 1 |
| LSS30 | 2.155466 | 2.703822 | 0.331711 |
| LSS30 | 3.342668 | 2.191624 | 0.270744 |
| LSS30 | 3.069492 | 3.630077 | 0.478304 |
| Iva30 | 1.043189 | 1.406393 | 0.780786 |
| Iva30 | 1.022429 | 1.201636 | 1.028114 |
| Iva30 | 1.02669 | 1.41814 | 0.806642 |
| LY30 | 1.618884 | 2.093621 | 0.183011 |
| LY30 | 1.94531 | 2.226843 | 0.192776 |
| LY30 | 1.933212 | 2.566852 | 0.313383 |

| $2^{-\Delta\Delta CT}$ / group | IL-6 | VCAM-1 | eNOS |
|---|---|---|---|
| LSS0 | 1 | 1 | 1 |
| LSS0 | 1 | 1 | 1 |
| LSS0 | 1 | 1 | 1 |
| LSS120 | 1.949359 | 1.982059 | 0.683494 |
| LSS120 | 1.523089 | 2.640846 | 0.596668 |
| LSS120 | 2.604488 | 1.876442 | 0.658383 |
| Iva0 | 1.140764 | 1.126619 | 0.961927 |
| Iva0 | 0.964598 | 1.414214 | 1.175276 |
| Iva0 | 1.583372 | 0.978742 | 0.983502 |
| Iva120 | 1.174462 | 1.057018 | 1.032399 |
| Iva120 | 1.000693 | 1.091264 | 1.046085 |
| Iva120 | 1.464086 | 1.036701 | 0.926588 |
