# Supplementary material for: Ivabradine Prevents Low Shear Stress Induced Endothelial Inflammation and Oxidative Stress via mTOR/eNOS Pathway
Source: PLoS One. 2016 Feb 18;11(2):e0149694. doi: 10.1371/journal.pone.0149694 (PMC4758626; doi:10.1371/journal.pone.0149694)
Supplement: S2 Table — (PDF) [file pone.0149694.s002.pdf]

group \ positive rate	DHE	DCF	IL-6	VCAM-1	eNOS-Thr495
LSS0	0.0308	0.064516	0.02135	0.039007	0.051282
LSS0	0.00877	0.046283	0.03508	0.030303	0.055555
LSS0	0.00658	0.050222	0.01379	0.03492	0.032967
LSS0	0.01685	0.046379	0.03614	0.02848	0.05
LSS0	0.02614	0.048298	0.04	0.046428	0.04301
LSS30	0.51786	0.677137	0.5	0.47445	0.0583333
LSS30	0.38583	0.584192	0.5955	0.63725	0.5
LSS30	0.42537	0.740668	0.44186	0.692307	0.451219
LSS30	0.4964	0.734375	0.51538	0.608108	0.4090909
LSS30	0.58462	0.538476	0.44339	0.474025	0.547297
Iva0	0.153846	0.045593	0.05147	0.04918	0.030303
Iva0	0.16129	0.063091	0.03529	0.038095	0.02325
Iva0	0.175439	0.062092	0.0318	0.045161	0.0510204
Iva0	0.194805	0.057399	0.01069	0.039173	0.0592178
Iva0	0.1	0.043786	0.01148	0.044982	0.0429764
Iva30	0.029703	0.11093	0.055172	0.075555	0.0628019
Iva30	0.04	0.110106	0.071428	0.052631	0.084501
Iva30	0.075269	0.130014	0.079681	0.050691	0.067873
Iva30	0.074766	0.123858	0.065149	0.051849	0.059248
Iva30	0.048544	0.122385	0.05873	0.047103	0.0528194

group \ positive rate	DHE	DCF	IL-6	VCAM-1	eNOS-Thr495
LSS0	0.0308	0.064516	0.02135	0.039007	0.051282
LSS0	0.00877	0.046283	0.03508	0.030303	0.055555
LSS0	0.00658	0.050222	0.01379	0.03492	0.032967
LSS0	0.01685	0.046379	0.03614	0.02848	0.05
LSS0	0.02614	0.048298	0.04	0.046428	0.04301
LSS30	0.51786	0.677137	0.5	0.47445	0.0583333
LSS30	0.38583	0.584192	0.5955	0.63725	0.5
LSS30	0.42537	0.740668	0.44186	0.692307	0.451219
LSS30	0.4964	0.734375	0.51538	0.608108	0.4090909
LSS30	0.58462	0.538476	0.44339	0.474025	0.547297
Iva30	0.029703	0.11093	0.055172	0.075555	0.0628019
Iva30	0.04	0.110106	0.071428	0.052631	0.084501
Iva30	0.075269	0.130014	0.079681	0.050691	0.067873
Iva30	0.074766	0.123858	0.065149	0.051849	0.059248
Iva30	0.048544	0.122385	0.05873	0.047103	0.0528194
LY30	0.5709	0.487106	0.53431	0.683168	0.436274
LY30	0.4811	0.478455	0.542857	0.523076	0.446511
LY30	0.6861	0.458333	0.515384	0.449152	0.464646
LY30	0.5188	0.487639	0.513849	0.539185	0.478538
LY30	0.3953	0.469729	0.518375	0.622874	0.4375892

positive rate / group	DHE	DCF	IL-6	VCAM-1	eNOS-Thr495
LSS0	0.0308	0.064516	0.02135	0.039007	0.051282
LSS0	0.00877	0.046283	0.03508	0.030303	0.055555
LSS0	0.00658	0.050222	0.01379	0.03492	0.032967
LSS0	0.01685	0.046379	0.03614	0.02848	0.05
LSS0	0.02614	0.048298	0.04	0.046428	0.04301
LSS120	0.432767	0.661184	0.571429	0.556818	0.0583333
LSS120	0.442219	0.664948	0.692913	0.608396	0.5
LSS120	0.451074	0.670947	0.647482	0.639456	0.451219
LSS120	0.472313	0.659275	0.566176	0.543269	0.4090909
LSS120	0.467446	0.672875	0.708661	0.55	0.547297
Iva0	0.033846	0.045593	0.04918	0.04918	0.030303
Iva0	0.03129	0.063091	0.038095	0.038095	0.02325
Iva0	0.025439	0.062092	0.045161	0.045161	0.0510204
Iva0	0.034805	0.057399	0.039173	0.039173	0.0592178
Iva0	0.02	0.043786	0.044982	0.044982	0.0429764
Iva120	0.112637	0.134615	0.082949	0.132353	0.0628019
Iva120	0.120915	0.126453	0.098712	0.096491	0.084501
Iva120	0.106101	0.130802	0.011111	0.128755	0.067873
Iva120	0.131222	0.128765	0.106838	0.117371	0.059248
Iva120	0.138539	0.137476	0.095477	0.123894	0.0528194
