# Supplementary material for: Ivabradine Prevents Low Shear Stress Induced Endothelial Inflammation and Oxidative Stress via mTOR/eNOS Pathway
Source: PLoS One. 2016 Feb 18;11(2):e0149694. doi: 10.1371/journal.pone.0149694 (PMC4758626; doi:10.1371/journal.pone.0149694)
Supplement: S3 Table — (PDF) [file pone.0149694.s003.pdf]

eNOS-Thr495 / group	Mean	SD	N
LSS0	0.44707	0.415133	3
LSS5	1.62141	0.440732	3
LSS15	1.92779	0.679016	3
LSS30	2.20396	0.48966	3
LSS60	1.77627	0.465466	3
LSS120	1.90368	0.117605	3

p70S6K / group	Mean	SD	N
LSS0	0.367572	0.094126	3
LSS5	0.796154	0.184659	3
LSS15	1.004122	0.307339	3
LSS30	0.85769	0.233371	3
LSS60	0.423026	0.039829	3
LSS120	0.262383	0.121685	3

Akt-Ser473 / group	Mean	SD	N
LSS0	2.13984	0.380339	3
LSS5	1.41354	0.336529	3
LSS15	1.44019	0.354117	3
LSS30	1.87183	0.344326	3
LSS60	1.45029	0.236104	3
LSS120	2.06781	0.426976	3

rictor / group	Mean	SD	N
LSS0	1.838192	0.711462	3
LSS5	1.131999	0.492859	3
LSS15	0.955771	0.460782	3
LSS30	0.603016	0.312299	3
LSS60	0.720377	0.331103	3
LSS120	0.59699	0.28975	3

S6RP / group	Mean	SD	N
LSS0	1.09609	0.167971	3
LSS5	1.3518	0.196438	3
LSS15	1.59734	0.221885	3
LSS30	1.41496	0.277374	3
LSS60	1.12461	0.289305	3
LSS120	0.65011	0.195668	3

raptor / group	Mean	SD	N
LSS0	0.134131	0.062236	3
LSS5	0.196258	0.049744	3
LSS15	1.436064	0.262562	3
LSS30	1.422383	0.203643	3
LSS60	0.650205	0.072318	3
LSS120	0.699628	0.101285	3

p70S6K / group	Mean	SD	N
LSS0	0.68871	0.147735	3
Iva0	0.5399	0.185534	3
LSS30	1.64788	0.315498	3
Iva30	0.38658	0.130383	3

rictor / group	Mean	SD	N
LSS0	1.291165	0.274109	3
Iva0	1.051972	0.17112	3
LSS30	0.182833	0.08975	3
Iva30	1.346067	0.2091	3

S6RP / group	Mean	SD	N
LSS0	0.24292	0.063246	3
Iva0	0.58346	0.133288	3
LSS30	1.28016	0.271819	3
Iva30	0.58102	0.110616	3

eNOS-Thr495 / group	Mean	SD	N
LSS0	0.486134	0.078159	3
Iva0	0.545473	0.097137	3
LSS30	1.397495	0.13834	3
Iva30	1.142658	0.175568	3

raptor / group	Mean	SD	N
LSS0	0.1815	0.10304	3
Iva0	0.59679	0.090838	3
LSS30	1.51033	0.194213	3
Iva30	0.69725	0.045503	3

Akt-Ser473 / group	Mean	SD	N
LSS0	1.445111	0.211138	3
Iva0	1.345494	0.123222	3
LSS30	1.005887	0.027636	3
Iva30	1.199221	0.074375	3

group \ rictor	Mean	SD	N
LSS0	0.55384	0.051004	3
Iva0	0.53699	0.066589	3
LSS120	0.10416	0.010572	3
Iva120	0.25655	0.019136	3

group \ eNOS-Thr495	Mean	SD	N
LSS0	0.153825	0.068492	3
Iva0	0.136464	0.067635	3
LSS120	0.72984	0.107356	3
Iva120	0.526113	0.094693	3

group \ Akt-Ser473	Mean	SD	N
LSS0	1.45153	0.179819	3
Iva0	1.37878	0.166557	3
LSS120	0.27412	0.077662	3
Iva120	1.20965	0.093877	3

group \ Akt-Ser473	Mean	SD	N
LSS0	1.80518	0.079358	3
LSS30	0.47745	0.201589	3
Iva30	1.78655	0.091666	3
LY30	0.579	0.170123	3

group \ p70S6K	Mean	SD	N
LSS0	0.441123	0.091589	3
LSS30	1.016074	0.091016	3
Iva30	0.510819	0.074092	3
LY30	1.212586	0.11849	3

group \ eNOS-Thr495	Mean	SD	N
LSS0	1.13381	0.069628	3
LSS30	2.32769	0.417334	3
Iva30	1.25346	0.283106	3
LY30	2.0078	0.463014	3

group \ rictor	Mean	SD	N
LSS0	0.554327	0.078955	3
LSS30	0.197009	0.050213	3
Iva30	0.807351	0.062418	3
LY30	0.277335	0.056082	3

group \ S6RP	Mean	SD	N
LSS0	0.31836	0.044745	3
LSS30	0.91998	0.053435	3
Iva30	0.5449	0.046483	3
LY30	0.62331	0.041556	3

group \ raptor	Mean	SD	N
LSS0	0.037474	0.003589	3
LSS30	0.672651	0.056251	3
Iva30	0.141555	0.004514	3
LY30	1.247958	0.016444	3
